# Inverse association between high-density lipoprotein cholesterol and bone mineral density in patients with diabetes results from a nationally representative survey

**DOI:** 10.1097/MD.0000000000049585

**Published:** 2026-07-17

**Authors:** Yi Tang, Xia Li, Jiewen Wei, Jiale Liu, Yunhao Wang, Hongxing Liao

**Affiliations:** a The First School of Clinical Medicine, Guangdong Medical University, Zhanjiang, China; b Department of Sports Medicine, Affiliated Meizhou Hospital of Shantou University Medical College, Meizhou, China; c Shantou University Medical College, Shantou, China; d Department of Hand Surgery, Huizhou Sixth People’s Hospital, Huizhou, China.

**Keywords:** bone mineral density, diabetes mellitus, high-density lipoprotein, NHANES, osteoporosis

## Abstract

Diabetes mellitus substantially increases the risk of osteoporosis and fractures, but the mechanisms remain unclear. Dyslipidemia, particularly the role of high-density lipoprotein (HDL), may influence bone mineral density (BMD), yet evidence in diabetic patients is limited. This study aimed to explore the association between HDL and BMD in US adults with diabetes using a nationally representative dataset. We analyzed data from 5 National Health and Nutrition Examination Survey cycles (2005–2018). A total of 5014 diabetic participants with available HDL and dual-energy X-ray absorptiometry (DXA)-measured BMD were included. Weighted multivariable linear regression models, smooth curve fitting, and 2-piecewise linear regression were employed to examine linear and nonlinear associations. Subgroup and sensitivity analyses were also conducted to assess robustness. Among 5014 participants (mean age 40.1 ± 31.2 years; 50.8% male), mean femoral neck and lumbar spine BMD were 0.81 and 1.03 g/cm^2^, respectively, with a mean HDL of 48.9 mg/dL. Higher HDL levels were inversely associated with BMD at both the femoral neck (β = −0.0011, 95% CI: −0.0015 to −0.0007) and lumbar spine (β = −0.0012, 95% CI: −0.0018 to −0.0007) after full adjustment. Participants in the highest HDL quartile had significantly lower BMD compared with those in the lowest quartile (femoral neck: −0.0294 g/cm^2^; lumbar spine: −0.0378 g/cm^2^). Smooth curve fitting indicated nonlinear, negative associations. Subgroup analyses revealed stronger inverse associations in men, participants with hypertension, those <60 years at the femoral neck, and those ≥60 years at the lumbar spine. Sensitivity analyses confirmed the robustness of the results. Higher HDL levels were consistently and independently associated with lower BMD in US adults with diabetes. These findings challenge the traditional view of HDL as universally protective, suggesting that in the diabetic context, elevated HDL may exert detrimental effects on bone health. Further longitudinal and mechanistic studies are warranted to clarify causal pathways and guide osteoporosis prevention strategies in this high-risk population.

## 1. Introduction

Diabetes mellitus is a common chronic metabolic disorder whose prevalence has risen dramatically worldwide over recent decades, posing a major public health challenge.^[1]^ Persistent hyperglycemia is well known to cause complications affecting the cardiovascular, renal, and nervous systems, but accumulating evidence indicates that skeletal health is also adversely impacted.^[2]^ Epidemiological studies consistently demonstrate that individuals with diabetes have a substantially higher risk of fractures than their nondiabetic counterparts, highlighting a heavy burden on public health.^[3]^ Because low BMD is a major determinant of fracture risk, understanding the factors influencing BMD in patients with diabetes is of great importance for the prevention of osteoporosis and fracture.

The mechanisms underlying diabetes-related bone alterations, however, remain incompletely elucidated. In addition to the direct effects of hyperglycemia, disturbances in lipid metabolism may play a pivotal role.^[3]^ High-density lipoprotein (HDL), a key antiatherogenic lipoprotein, has traditionally been considered protective against cardiovascular disease through reverse cholesterol transport, antioxidative, and anti-inflammatory functions.^[4]^ Yet, its role in bone metabolism remains controversial. Experimental studies suggest that HDL and its major apolipoprotein ApoA-I can promote osteoblast differentiation, inhibit osteoclastogenesis, and attenuate inflammation in bone tissue,^[5,6]^ while epidemiological evidence points to a possible positive association between HDL levels and BMD.^[7]^ In contrast, other studies have reported an inverse relationship, with elevated HDL levels linked to lower BMD, particularly in populations with metabolic disorders.^[8]^Importantly, most prior investigations have focused on general populations, and evidence specific to patients with diabetes – a group characterized by unique metabolic alterations – remains limited. Whether HDL exerts a protective or detrimental effect on bone health in this setting, and the extent to which diabetes-specific metabolic disturbances modify this association, remain unresolved questions.

The National Health and Nutrition Examination Survey (NHANES), a large, nationally representative program employing a stratified multistage probability sampling design, provides a valuable opportunity to address these gaps. NHANES collects detailed clinical, laboratory, and questionnaire data, including lipid profiles, diabetes status, and dual-energy X-ray absorptiometry (DXA)-derived BMD.^[9]^ Leveraging these data allows for systematic assessment of the relationship between HDL and BMD in patients with diabetes, while adjusting for multiple confounders. Therefore, in the present study, we aimed to investigate the association between HDL levels and BMD among US adults with diabetes, and to further examine its consistency across subgroups, with the goal of providing evidence to inform osteoporosis prevention and bone health management in this vulnerable population.

## 2. Materials and methods

### 2.1. Survey description

The NHANES, administered by the National Center for Health Statistics (NCHS), is a nationally representative cross-sectional survey designed to assess the health and nutritional status of the noninstitutionalized civilian US population. Employing a complex multistage stratified probability sampling design, NHANES collects biennial data, ensuring national representativeness.^[10]^ The NCHS Research Ethics Review Board approved all protocols. Written informed consent was obtained from all participants, with parental/guardian consent and child assent secured for minors. Comprehensive survey methods and data are publicly accessible (https://wwwn.cdc.gov/nchs/nhanes/). This analysis adhered to STROBE guidelines for observational studies.

### 2.2. Study population

**Figure 1.**
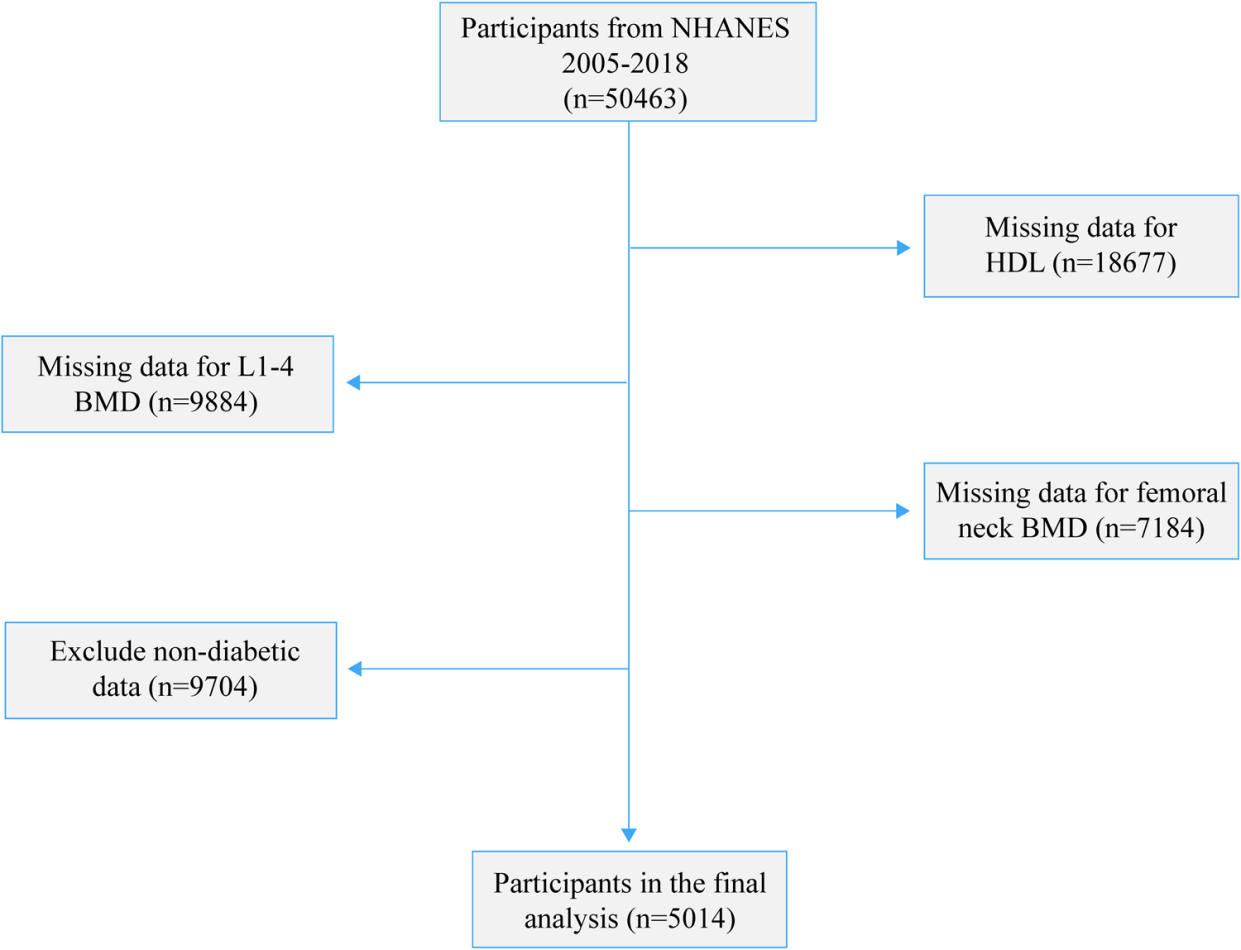
Participant flow diagram. BMD = bone mineral density, HDL = high-density lipoprotein, NHANES = National Health and Nutrition Examination Survey.

Data from 5 NHANES cycles (2005–2006,2007–2008,2009–2010,2013–2014,2017–2018) were analyzed. Exclusion criteria included: missing femoral neck BMD (n = 7184), lumbar spine (L1–L4) BMD (9884), HDL (n = 18,677), and nondiabetic patients (n = 9704). The final cohort comprised 5014 participants, as shown in Figure 1.

### 2.3. Exposure variables

Briefly, based on the information provided on the NHANES website, the HDL measurement was performed at the Lipid Laboratory, Johns Hopkins. Serum was collected for the detection of HDL. Apolipoprotein-B (apoB)-containing lipoproteins were removed by reaction with blocking reagents, rendering them nonreactive with enzymatic cholesterol reagents under the assay conditions. HDL levels were measured using polyethylene glycol-coupled cholesteryl esterase, cholesterol oxidase, and sulfated α-cyclodextrin in the presence of Mg^2+^. Detailed information about the measurement of HDL is accessible on the NHANES website.^[11]^

### 2.4. Outcomes

BMD was evaluated using dual-energy X-ray absorptiometry (DXA) scans. The sites of assessment included the femur neck and the lumbar spine (L1, L2, L3, L4). Health technologists who were certified radiology technologists conducted the DXA scans using a Hologic QDR 4500A instrument (Hologic, Inc., Bedford) and Apex software version 3.2. Further details of the DXA examination protocol are described in the Body Composition Procedures Manual provided on the NHANES website.^[12]^

### 2.5. Covariables

Demographic data included age, sex, race/ethnicity, education level, and the ratio of family income to the poverty threshold. Laboratory data consisted of total cholesterol concentration measured in serum. Information obtained from questionnaires included alcohol consumption status, defined as having consumed at least twelve alcoholic drinks in the past year; smoking history, defined as having smoked at least one hundred cigarettes during one’s lifetime; body mass index calculated from measured height and weight; and self-reported physician diagnoses of diabetes and hypertension.^[13]^ To account for the complex survey design, we applied the Full Sample Two-Year Mobile Examination Center Exam Weight (WTMEC2YR) recommended by NHANES analytic guidelines. Because 5 consecutive 2-year cycles were combined, the final analytic weight was calculated as one-fifth of the original WTMEC2YR value.^[14]^

### 2.6. Statistical analysis

All analyses were conducted among patients with diabetes, incorporating NHANES sampling weights, strata, and primary sampling units. Baseline characteristics were compared across HDL quartiles using weighted chi-square tests for categorical variables and linear regression for continuous variables. Weighted multivariable linear regression was used to evaluate the associations between HDL and BMD in 3 models: model 1 (unadjusted); model 2 (adjusted for age, sex, and race); and model 3 (fully adjusted for all covariates). We examined the nonlinear relationship between HDL and BMD using an estimating technique that combined a generalized additive model with a smoothing curve. Upon identifying nonlinearity, we adopted a recursive strategy to locate the inflection point within the connection between BMD and HDL. For a more detailed understanding of this nonlinear pattern, a biphasic linear regression model was applied around the identified inflection point. While the NHANES employs advanced sampling procedures to increase the representativeness and application of its findings, weighted and unweighted analyses may provide skewed results in some circumstances. We performed a sensitivity analysis with unweighted regression to revalidate our findings in this study. Subgroup analyses were then carried out to evaluate the data’s consistency and dependability. The statistical analyses were carried out using the software programs PackageR and EmpowerStats, which are accessible at http://www.r-project.org and http://www.empowerstats.com, respectively. It was determined that statistical significance was indicated by a *P* value <.05.

## 3. Results

### 3.1. Baseline characteristics

A total of 5014 participants with a mean age of 40.13 ± 31.17 years were included (50.81% male). The population comprised 36.41% non-Hispanic White, 22.35% Mexican American, 9.07% other Hispanic, 22.95% non-Hispanic Black, and 9.21% other races. The mean femoral neck BMD, lumbar spine BMD, and HDL were 0.81 (0.16) g/cm^2^, 1.03 (0.17) g/cm^2^, and 48.86 (14.38) mg/dL, respectively. Baseline characteristics differed significantly across HDL quartiles (*P* < .05). Participants in the highest quartile were more likely to be female, non-Hispanic White, older, better educated, wealthier, physically active, and to have higher cholesterol, but had lower BMI, lower smoking and alcohol use, less hypertension, and lower femoral neck and lumbar spine BMD. All clinical characteristics of the patients are listed by HDL quartile in Table 1.

**Table 1:** Weighted baseline characteristics by HDL Quartiles in US adults (NHANES 2005–2018).

HDL	Q1 (11–39)	Q2 (39–46)	Q3 (46–56)	Q4 (56–76)	*P* value
Age (yr)	57.31 ± 14.31	57.69 ± 14.87	58.06 ± 15.68	60.23 ± 15.54	<.0001
Sex (%)					<.0001
Male	71.04	55.08	47.07	29.36	
Female	28.96	44.92	52.93	70.67	
Race/ethnicity (%)					<.0001
Mexican American	7.65	10.57	8.80	8.47	
Other Hispanic	4.22	5.90	4.82	5.24	
Non-Hispanic White	70.49	63.29	63.51	57.44	
Non-Hispanic Black	8.85	12.44	14.72	19.30	
Other Race – Including Multi-Racial	8.79	7.80	8.15	9.55	
PIR	2.80 ± 1.59	2.77 ± 1.64	2.84 ± 1.63	2.91 ± 1.56	<.0001
Education level (%)					.0253
Less than high school	24.98	26.25	21.22	20.19	
High school	25.78	25.99	24.23	27.09	
More than high school	49.24	47.76	54.55	52.72	
Marital status					<.0001
Married/Live with partner	66.62	67.22	65.00	58.04	
Never married	9.72	7.35	7.99	8.53	
Separated/Divorced/Widowed	23.66	25.43	27.01	33.43	
Smoked at least 100 cigarettes in life (%)					<.0001
Yes	61.33	52.44	44.34	42.03	
No	38.67	47.56	55.64	57.97	
Alcoholic ≥ 4 drinks/d					<.0001
Yes	25.00	21.06	16.23	14.69	
No	75.00	78.64	83.77	85.31	
Moderate activity					<.0001
Yes	35.53	31.04	43.23	36.52	
No	64.47	69.96	32.53	63.48	
Hypertension (%)					.0329
Yes	69.73	64.72	63.05	63.62	
No	30.12	34.84	36.95	36.38	
BMI (kg/ m^2^)	34.23 ± 7.57	33.55 ± 7.64	32.29 ± 7.52	30.70 ± 7.58	<.0001
Cholesterol (mg/dL)	174.93 ± 50.23	180.19 ± 48.35	184.35 ± 43.73	190.41 ± 42.22	<.0001
Femoral neck BMD (g/cm^2^)	0.83 ± 0.14	0.82 ± 0.16	0.79 ± 0.16	0.77 ± 0.15	<.0001
L1–L4 BMD (g/cm^2^)	1.08 ± 0.16	1.06 ± 0.17	1.05 ± 0.16	1.02 ± 0.18	<.0001

Data presented as mean ± SD or weighted percentage. *P* values from weighted linear regression (continuous) or χ^2^ tests (categorical).

BMD = bone mineral density, HDL = high-density lipoprotein, PIR = poverty-to-income ratio.

### 3.2. Association between the HDL and BMD among patients with diabetes

As shown in Table 2, HDL levels were inversely associated with BMD at both skeletal sites. In the unadjusted model, HDL was negatively linked with BMD (β = −0.0017, 95% CI: −0.0021 to −0.0013 for femoral neck BMD; β = −0.0016, 95% CI: −0.0021 to −0.0011 for L1–L4 BMD; both *P* < .0001). These associations persisted after adjustment for age, sex, and race/ethnicity (model 2) and remained robust following further adjustment for socioeconomic, lifestyle, and clinical factors (model 3: β = −0.0011, 95% CI: −0.0015 to −0.0007 for femoral neck BMD; β = −0.0012, 95% CI: −0.0018 to −0.0007 for L1–L4 BMD; both *P* < .0001).When HDL was analyzed in quartiles, a graded inverse trend was evident (*P* for trend < .05). In the fully adjusted model, participants in the highest HDL quartile had significantly lower BMD compared with those in the lowest quartile, with a reduction of 0.0294 g/cm^2^ at the femoral neck (95% CI: −0.0456 to −0.0132) and 0.0378 g/cm^2^ at the lumbar spine (95% CI: −0.0598 to −0.0159).

**Table 2:** Association between HDL and bone mineral density in diabetes patients.

Exposure	Model 1 β (95% CI)	Model 2 β (95% CI)	Model 3 β (95% CI)
Femoral neck BMD (g/cm^2^)			
HDL	−0.0017 (−0.0021, −0.0013)	−0.0013 (−0.0017, −0.0009)	−0.0011 (−0.0015, −0.0007)
HDL categories			
Q1	Reference	Reference	Reference
Q2	−0.0105 (−0.0226, 0.0056)	−0.0048 (−0.0194, 0.0097)	0.0002 (−0.0152, 0.0155)
Q3	−0.0409 (−0.0567, −0.0252)	−0.0315 (−0.0459, −0.0171)	−0.0302 (−0.0456, −0.0149)
Q4	−0.0578 (−0.0737, −0.0418)	−0.0352 (−0.0504, −0.0200)	−0.0294 (−0.0456, −0.0132)
*P*-value for tend	<.05	<.05	<.05
L1–L4 BMD (g/cm^2^)			
HDL	−0.0016 (−0.0021, −0.0011)	−0.0013 (−0.0018, −0.0007)	−0.0012 (−0.0018, −0.0007)
HDL categories			
Q1	Reference	Reference	Reference
Q2	−0.0175 (−0.0387, 0.0038)	−0.0076 (−0.0285, 0.0133)	−0.0037 (−0.0252, 0.0179)
Q3	−0.0278 (−0.0482, −0.0074)	−0.0218 (−0.0420, −0.0016)	−0.0259 (−0.0469, −0.0048)
Q4	−0.0582 (−0.0785, −0.0378)	−0.0412 (−0.0622, −0.0202)	−0.0378 (−0.0598, −0.0159)
*P*-value for tend	<.05	<.05	<.05

Model 1: unadjusted. Model 2: adjusted for age, sex, race/ethnicity. Model 3: additionally adjusted for PIR, education, marital status, smoking, alcohol intake, hypertension, MI, and cholesterol.

BMD = bone mineral density, CI = confidence interval, HDL = high-density lipoprotein, MI = myocardial infarction, PIR = poverty-to-income ratio.

### 3.3. A nonlinear association between HDL and BMD among patients with diabetes

**Figure 2.**
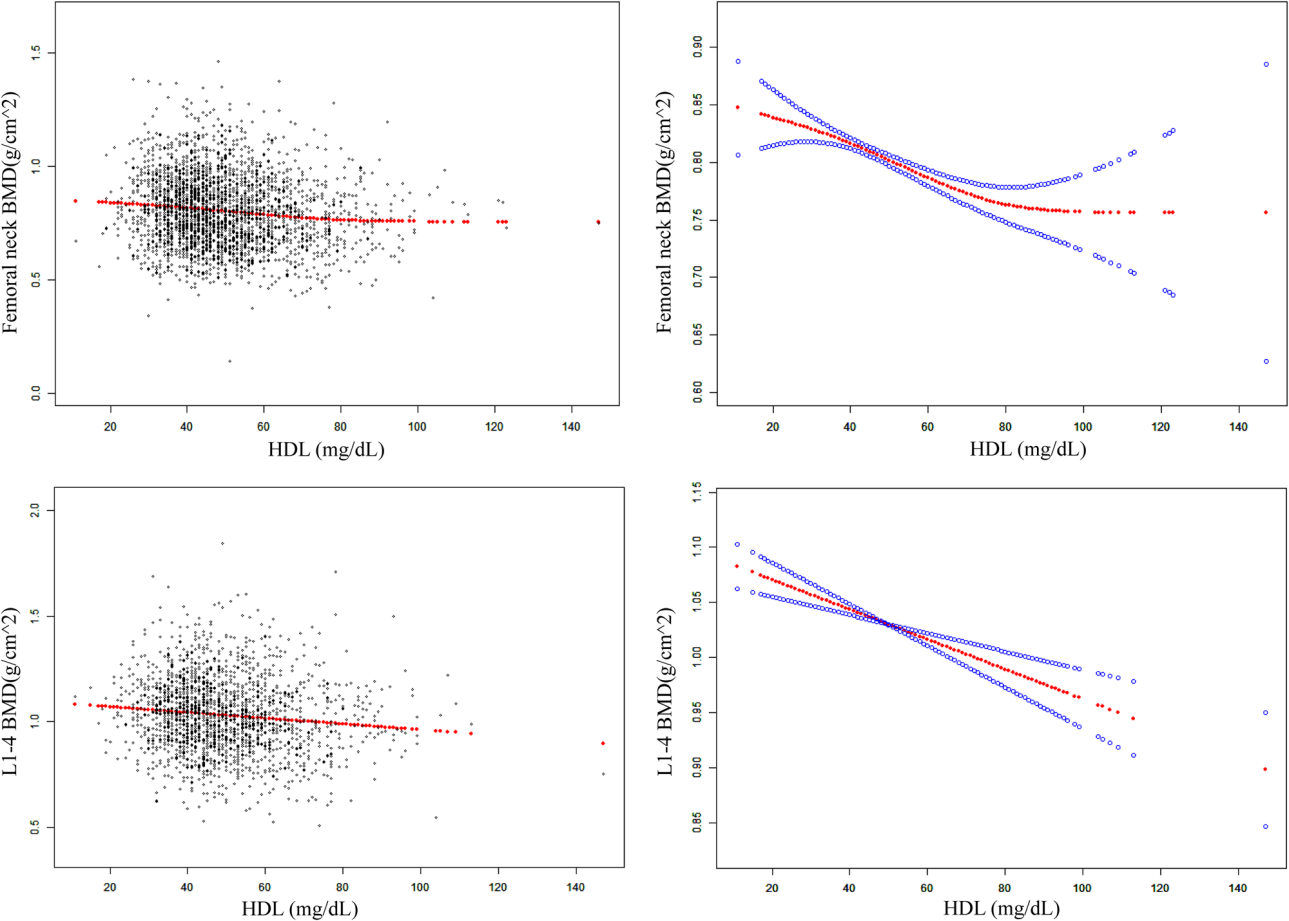
Association between HDL and BMD in diabetes patients. (A, B) Association between HDL and femoral neck BMD; (C, D) association between HDL and L1–L4 BMD; (A, C) each black point represents a sample; and (B, D) the solid red line represents the smooth curve fit between variables. Blue bands represent the 95% confidence interval from the fit. Age, sex, race/ethnicity, BMI, PIR, education, marital status, smoking, alcohol consumption, moderate physical activity, hypertension, and cholesterol were adjusted. BMI = body mass index, MD = bone mineral density, HDL = high-density lipoprotein, PIR = poverty-to-income ratio.

This study employed smooth curve fitting and 2-segment linear regression models to examine the nonlinear relationship between HDL and BMD of the femoral neck and L1–L4. The findings underscored a nonlinear link, establishing a negative correlation between HDL and BMD at both skeletal sites, as illustrated in Figure 2.

### 3.4. Sensitivity analysis

Similarly, sensitivity analyses using unweighted logistic analyses showed that individuals with the highest quartile of HDL had a lower BMD than those with the lowest quartile of HDL, according to the different models: model 1 (β = −0.0475, 95% CI: −0.0638 to −0.0313 for femoral neck BMD; β = −0.0498, 95% CI: −0.0713 to −0.0282 for L1–L4 BMD; both *P* < .0001), model 2 (β = −0.0321, 95% CI: −0.0474 to −0.0168 for femoral neck BMD; β = −0.0365, 95% CI: −0.0581 to −0.0149 for L1–L4 BMD; both *P* < .0001), and model 3 (β = −0.0326, 95% CI: −0.0488 to −0.0165 for femoral neck BMD; β = −0.0390, 95% CI: −0.0615 to −0.0165 for L1–L4 BMD; both *P* < .0001; Table 3). HDL and BMD appear to consistently correlate negatively, according to these findings.

**Table 3:** Unweighted logistic regression analysis on the association between HDL and BMD in sensitive analysis.

Exposure	Model 1 β (95% CI)	Model 2 β (95% CI)	Model 3 β (95% CI)
Femoral neck BMD (g/cm^2^)			
HDL	−0.0013 (−0.0017, −0.0009)	−0.0010 (−0.0013, −0.0006)	−0.0010 (−0.0013, −0.0006)
HDL categories			
Q1	Reference	Reference	Reference
Q2	−0.0090 (−0.0256, 0.0077)	−0.0084 (−0.0233, 0.0065)	−0.0041 (−0.0198, 0.0115)
Q3	−0.0269 (−0.0435, −0.0103)	−0.0196 (−0.0347, −0.0046)	−0.0212 (−0.0370, −0.0053)
Q4	−0.0475 (−0.0638, −0.0313)	−0.0321 (−0.0474, −0.0168)	−0.0326 (−0.0488, −0.0165)
*P*-value for tend	<.05	<.05	<.05
L1–L4 BMD (g/cm^2^)			
HDL	−0.0014 (−0.0019, −0.0008)	−0.0011 (−0.0017, −0.0006)	−0.0012 (−0.0018, −0.0006)
HDL categories			
Q1	Reference	Reference	Reference
Q2	−0.0092 (−0.0316, 0.0132)	−0.0019 (−0.0234, 0.0197)	−0.0025 (−0.0199, 0.0248)
Q3	−0.0212 (−0.0434, −0.0010)	−0.0160 (−0.0376, −0.0057)	−0.0169 (−0.0393, −0.0055)
Q4	−0.0498 (−0.0713, −0.0282)	−0.0365 (−0.0581, −0.0149)	−0.0390 (−0.0615, −0.0165)
*P*-value for tend	<.05	<.05	<.05

Model 1: unadjusted. Model 2: adjusted for age, sex, race/ethnicity. Model 3: additionally adjusted for PIR, education, marital status, smoking, alcohol intake, hypertension, MI, and cholesterol.

BMD = bone mineral density, CI = confidence interval, HDL = high-density lipoprotein.

### 3.5. Subgroup analysis

Further subgroup analyses were conducted to explore the robustness of the association between HDL and BMD across different demographic and clinical characteristics (Table 4). After adjustment for potential confounders, the effect estimates remained relatively stable across most subgroups. No significant interaction effects were observed for race/ethnicity, BMI, smoking, alcohol consumption, or income level. However, stratification by sex, age, physical activity, and hypertension revealed significant heterogeneity. The negative association was stronger among men than women for both femoral neck and lumbar spine BMD, with significant interaction terms (*P* for interaction = .0071 and < .0001, respectively). Similarly, participants younger than 60 years and those aged ≥60 years showed differential effects, with more pronounced associations in younger individuals for femoral neck BMD and in older individuals for lumbar spine BMD (*P* for interaction = .0111 and .0469, respectively). Moreover, the association was significantly stronger among individuals without moderate activity (*P* for interaction = .0004 for femoral neck; .0361 for lumbar spine) and among those with hypertension (*P* for interaction = .0007 and .0049, respectively).

**Table 4:** Subgroup Analysis of HDL-BMD Associations in diabetes patients stratified by sex, race/ethnicity, and comorbidity status.

Characteristics	β (95% CI)	*P* for interaction	
Femoral neck BMD (g/cm^2^)			
Subgroup analysis stratified by sex			.0071
Men	−0.0018 (−0.0023, −0.0012)		
Women	−0.0007 (−0.0012, −0.0002)		
Subgroup analysis stratified by race/ethnicity			.8837
Mexican American	−0.0023 (−0.0038, −0.0009)		
Other Hispanic	−0.0014 (−0.0032,0.0004)		
Non-Hispanic White	−0.0020 (−0.0025, −0.0015)		
Non-Hispanic Black	−0.0019 (−0.0029, −0.0010)		
Other Race – including multi-racial	−0.0025 (−0.0037, −0.0012)		
Subgroup analysis stratified by age			.0111
<60	−0.0015 (−0.0022, −0.0007)		
≥60	−0.0013 (−0.0017, −0.0008)		
Subgroup analysis stratified by BMI			.0664
<25	−0.0009 (−0.0015, −0.0003)		
25–30	−0.0009 (−0.0016, −0.0002)		
>30	−0.0016 (−0.0024, −0.0008)		
Subgroup analysis stratified by PIR			.6972
<1.3	−0.0010 (−0.0019, −0.0001)		
1.3–3.5	−0.0015 (−0.0022, −0.0007)		
>3.5	−0.0012 (−0.0017, −0.0006)		
Subgroup analysis stratified by moderate activity			.0004
Yes	−0.0017 (−0.0029, −0.0005)		
No	−0.0032 (−0.0045, −0.0018)		
Subgroup analysis stratified by smoking			.1109
Yes	−0.0040 (−0.0090, −0.0171)		
No	−0.0012 (−0.0018, −0.0007)		
Subgroup analysis stratified by alcohol			.2988
Yes	−0.0014 (−0.0020, −0.0008)		
No	−0.0007 (−0.0019, −0.0005)		
Subgroup analysis stratified by hypertension			.0007
Yes	−0.0017 (−0.0022, −0.0012)		
No	−0.0013 (−0.0020, −0.0007)		
L1–4 BMD (g/cm^2^)			
Subgroup analysis stratified by sex			<.0001
Men	−0.0015 (−0.0022, −0.0008)		
Women	−0.0010 (−0.0018, −0.0002)		
Subgroup analysis stratified by race/ethnicity			.9495
Mexican American	−0.0007 (−0.0024, −0.0010)		
Other Hispanic	−0.0015 (−0.0038,0.0008)		
Non-Hispanic White	−0.0012 (−0.0019, −0.0005)		
Non-Hispanic Black	−0.0014 (−0.0026, −0.0001)		
Other Race – including multi-racial	−0.0016 (−0.0032, −0.0000)		
Subgroup analysis stratified by age			.0469
<60	−0.0012 (−0.0019, −0.0004)		
≥60	−0.0019 (−0.0026, −0.00012)		
Subgroup analysis stratified by BMI			.1516
<25	−0.0020 (−0.0025, −0.0015)		
25–30	−0.0019 (−0.0029, −0.0010)		
>30	−0.0025 (−0.0037, −0.0012)		
Subgroup analysis stratified by PIR			.4680
<1.3	−0.0014 (−0.0027, −0.0000)		
1.3–3.5	−0.0020 (−0.0030, −0.0009)		
>3.5	−0.0012 (−0.0019, −0.0004)		
Subgroup analysis stratified by moderate activity			.0361
Yes	−0.0006 (−0.0019, −0.0003)		
No	−0.0017 (−0.0033, −0.0001)		
Subgroup analysis stratified by smoking			.8970
Yes	−0.0012 (−0.0019, −0.0005)		
No	−0.0011 (−0.0018, −0.0004)		
Subgroup analysis stratified by alcohol			.2988
Yes	−0.0014 (−0.0020, −0.0008)		
No	−0.0007 (−0.0019, −0.0005)		
Subgroup analysis stratified by hypertension			.0049
Yes	−0.0010 (−0.0016, −0.0003)		
No	−0.0009 (−0.0017, −0.0000)		

Fully adjusted model 3 covariates applied except for the stratification variable. Interaction *P* values test heterogeneity across subgroups.

BMD = bone mineral density, BMI = body mass index, CI = confidence interval, PIR = poverty-to-income ratio.

## 4. Discussion

In this nationally representative sample of US adults with diabetes, we found that higher HDL levels were consistently associated with lower BMD at both the femoral neck and lumbar spine. These associations remained robust after adjustment for multiple demographic, socioeconomic, lifestyle, and clinical covariates, and were further confirmed in sensitivity analyses. Subgroup analyses revealed effect modification by sex, age, physical activity, and hypertension, suggesting that the detrimental association of HDL with BMD may be more pronounced in specific diabetic subpopulations.

Our findings add to the growing but controversial literature regarding the role of HDL in bone metabolism. Previous epidemiological studies have yielded conflicting results. Several studies reported that higher HDL was associated with greater BMD or lower fracture risk, supporting the traditional protective role of HDL in bone health.^[7]^ Conversely, other investigations found an inverse relationship, with elevated HDL linked to reduced BMD, particularly among postmenopausal women or individuals with metabolic disorders.^[8]^ The current study provides important evidence in diabetic patients, a population with unique metabolic disturbances, indicating that HDL may not exert its classical protective effects on bone in this context.

To further interpret these findings, it is essential to consider the broader metabolic context, particularly the role of obesity and body mass index (BMI). Traditionally, a higher BMI has been regarded as protective for bone mineral density (BMD), primarily through increased mechanical loading that stimulates osteogenesis.^[15]^ However, this paradigm may not hold true in metabolically dysregulated conditions such as obesity and diabetes. Accumulating evidence indicates that elevated BMI in these populations does not necessarily confer skeletal protection and may instead be associated with impaired bone quality despite preserved or even increased BMD.^[16]^ Obesity is characterized by chronic low-grade inflammation, insulin resistance, and adipose tissue dysfunction, which together disrupt bone remodeling by enhancing osteoclastogenesis, suppressing osteoblast differentiation, and altering adipokine secretion (e.g., leptin and adiponectin).^[17]^ In addition, increased marrow adiposity promotes mesenchymal stem cell differentiation toward adipogenesis at the expense of osteogenesis, thereby compromising bone microarchitecture, while the “vitamin D dilution effect” may further impair calcium homeostasis.^[18]^ These adverse metabolic and endocrine alterations are often amplified in patients with diabetes, where hyperglycemia, advanced glycation end-products (AGEs), and oxidative stress can deteriorate collagen properties and bone material strength, leading to increased skeletal fragility independent of BMD.^[19]^ This may explain the paradoxical phenomenon whereby individuals with diabetes exhibit normal or elevated BMD but remain at higher risk of fractures. Furthermore, higher BMI is frequently associated with reduced physical performance and increased fall risk, further exacerbating fracture susceptibility. Taken together, these findings suggest that BMI should not be interpreted as a simple protective factor for bone health in metabolically compromised populations, and that bone quality, microarchitecture, and metabolic status should be considered collectively when evaluating fracture risk. Notably, these mechanisms may also partially underlie the observed inverse association between HDL-C and BMD in the present study, as HDL metabolism is closely linked to systemic inflammation, insulin resistance, and lipid homeostasis; in diabetes, dysfunctional HDL may lose its anti-inflammatory and antioxidative properties, thereby contributing to an unfavorable bone microenvironment despite elevated circulating levels.^[20]^

Beyond HDL-C, the relationship between lipid metabolism and bone health is complex and involves multiple lipid-related biomarkers. For example, triglycerides (TG) have shown inconsistent associations with BMD in previous studies. Some studies have reported a positive association, whereas others have found inverse or null relationships, suggesting that the link between TG and bone metabolism is complex and potentially context-dependent.^[7]^ Mechanistically, elevated TG levels are often associated with insulin resistance, chronic low-grade inflammation, and lipotoxicity, all of which may adversely affect bone remodeling by impairing osteoblast function and promoting osteoclast activity.^[21]^ In particular, excess circulating lipids can induce oxidative stress and disrupt mesenchymal stem cell differentiation, favoring adipogenesis over osteogenesis. However, in patients with diabetes, the relationship between TG and BMD appears to be even more complex and may be modified by multiple factors, including age, obesity status, menopausal state, and the presence of metabolic syndrome.^[19]^ These interacting factors may partly explain the inconsistent findings reported across studies. In the present study, we primarily focused on HDL-C as the exposure of interest; however, given the multifaceted role of lipid metabolism in bone biology, future studies should incorporate a broader lipid profile, including TG, LDL-C, apolipoprotein A-1 (ApoA-1), and apolipoprotein B (ApoB), to provide a more comprehensive understanding of the interplay between lipid metabolism and skeletal health.^[22]^

Among these lipid-related biomarkers, HDL and its principal apolipoprotein, ApoA-1, are of particular interest due to their central roles in lipid transport and their potential regulatory effects on bone metabolism. The relationship between HDL-C and apolipoprotein A-1 (ApoA-1) may provide additional insight into the observed association between lipid metabolism and bone health. ApoA-1 is the major structural and functional protein component of HDL and is generally positively correlated with circulating HDL-C levels.^[23]^ Emerging evidence suggests that ApoA-1 may exert beneficial effects on bone metabolism by promoting osteoblast differentiation and inhibiting osteoclast activity.^[24]^ However, in the context of diabetes, the functionality of HDL and its associated proteins, including ApoA-1, may be impaired. Glycation, oxidative modification, and chronic inflammation can lead to the formation of dysfunctional HDL, which exhibits reduced anti-inflammatory and antioxidative capacity.^[25]^ Under such conditions, elevated circulating HDL-C levels may not necessarily reflect preserved biological function, and therefore may not confer expected protective effects on bone. This concept may partly explain the inverse association observed between HDL-C and BMD in the present study. Future studies should incorporate a more comprehensive evaluation of lipid metabolism, including HDL functionality, ApoA-1, and other apolipoproteins, to better elucidate their roles in bone health and fracture risk, particularly in patients with diabetes.^[26]^

The subgroup findings in our study are noteworthy. The negative association between HD and BMD was stronger among men, participants with hypertension, those younger than 60 years for femoral neck BMD, and those older than 60 years for lumbar spine BMD. These results suggest sex- and age-specific differences in the lipid–bone relationship, which may reflect hormonal influences, differences in fat distribution, or cumulative vascular burden. Reduced physical activity also amplified the association, indicating that lifestyle factors may modify the impact of HDL on bone.^[27,28]^

This study has several strengths. We leveraged a large, nationally representative dataset with rigorous quality control, standardized measurements of HDL and BMD, and detailed covariate adjustment. Moreover, the consistency across weighted, unweighted, and subgroup analyses strengthens the robustness of our findings. However, some limitations should be acknowledged. First, due to the cross-sectional design, causal relationships cannot be inferred. Second, residual confounding by unmeasured factors, such as vitamin D status, sex hormones, or medications (e.g., statins, bisphosphonates), cannot be excluded. Third, BMD measured by DXA does not capture bone quality or microarchitecture, which also contribute to fracture risk. Finally, the observational nature of NHANES precludes direct mechanistic exploration.

Despite these limitations, our findings have important clinical implications. Given the high burden of osteoporosis and fracture among patients with diabetes,^[3]^ identifying modifiable metabolic factors related to bone health is critical. The inverse association between HDL and BMD highlights the complexity of lipid–bone interactions and suggests that HDL may not universally confer protection across all tissues, particularly under diabetic conditions. Future longitudinal studies and mechanistic investigations are needed to elucidate the causal direction and underlying pathways, and to determine whether interventions targeting lipid metabolism can improve skeletal health in patients with diabetes.

## 5. Conclusion

In conclusion, this nationally representative study of US adults with diabetes demonstrated that higher HDL levels were consistently and independently associated with lower BMD at both the femoral neck and lumbar spine. These associations remained robust across multiple sensitivity analyses and were more pronounced in specific subgroups, including men, individuals with hypertension, younger participants for femoral neck BMD, and older participants for lumbar spine BMD. Our findings highlight the complexity of the relationship between lipid metabolism and skeletal health in the context of diabetes and suggest that elevated HDL, traditionally considered protective in cardiovascular disease, may not exert the same beneficial effects on bone. Given the high burden of osteoporosis and fracture risk in diabetic populations, these results underscore the need for further longitudinal and mechanistic studies to clarify causal pathways and to explore whether interventions targeting lipid metabolism can contribute to improved bone health outcomes in this vulnerable group.

## Author contributions

**Conceptualization:** Yi Tang, Xia Li.

**Data curation:** Xia Li, Yunhao Wang.

**Formal analysis:** Xia Li, Jiewen Wei, Yunhao Wang.

**Funding acquisition:** Jiewen Wei, Yunhao Wang.

**Investigation:** Jiale Liu, Jiewen Wei

**Methodology:** Jiewen Wei

**Project administration:** Jiewen Wei.

**Resources:** Jiewen Wei.

**Software:** Xia Li, Jiewen Wei.

**Supervision:** Jiewen Wei, Hongxing Liao.

**Validation:** Xia Li, Jiewen Wei.

**Visualization:** Jiewen Wei.

**Writing – original draft:** Jiewen Wei, Yunhao Wang.

**Writing – review & editing:** Jiewen Wei, Yunhao Wang.
